# Applications of *Lactobacillus rhamnosus* Spent Culture Supernatant in Cosmetic Antioxidation, Whitening and Moisture Retention Applications

**DOI:** 10.3390/molecules181114161

**Published:** 2013-11-15

**Authors:** Cheng-Chih Tsai, Chin-Feng Chan, Wen-Ying Huang, Jin-Seng Lin, Patty Chan, Ho-Yen Liu, Yung-Sheng Lin

**Affiliations:** 1 Department of Food Science and Technology, Hungkuang University, Taichung 43302, Taiwan; E-Mail: tsaicc@sunrise.hk.edu.tw; 2 Department of Applied Cosmetology and Master Program of Cosmetic Science, Hungkuang University, Taichung 43302, Taiwan; E-Mails: cfchan@sunrise.hk.edu.tw (C.-F.C.); beca690420@hk.edu.tw (W.-Y.H.); jinsenglin@gmail.com (J.-S.L.); ving127@gmail.com (P.C.); wenyenliu@yahoo.com.tw (H.-Y.L.)

**Keywords:** *Lactobacillus rhamnosus*, antioxidation, whitening, moisture retention, tyrosinase

## Abstract

This study was aimed at investigating the antioxidant, whitening, and moisture-retention properties of *Lactobacillus rhamnosus* spent culture supernatant (Lr-SCS) in cosmetic applications. Results reveal that Lr-SCS effectively and gradually scavenges 1,1-diphenyl-2-picrylhydrazyl as well as 2,2-azino-bis-(3-ethylbenzothiazoline-6-sulfonic acid) radical cations, and increases reducing power in a dose-dependent manner. Additionally, Lr-SCS can also suppress tyrosinase activity *in vitro* and effectively promote moisture retention. Heat treatment at 100 °C for 30 min does not influence the functions of Lr-SCS. We conclude that Lr-SCS can be used effectively in skin care cosmetics.

## 1. Introduction

The World Health Organization defines probiotics as live microorganisms conferring a health benefit on the host when administered in adequate amounts. Probiotic bacterial cultures modulate the growth of intestinal microbiota, potentially suppressing harmful bacteria and reinforcing the body’s natural defense mechanisms. There is currently an abundance of evidence on the positive effects of probiotics on human health [1,2,3,4,5]. Probiotics are typically bacteria of the species *Lactobacillus* or *Bifidobacterium* [6], which are natural components of the gut microbiota and are thus able to survive in the gut. Among lactic acid bacteria (LAB), members of the genus *Lactobacillus* are generally regarded as non-pathogenic because of their long history of safe use as health-promoting organisms in numerous fermented food products—e.g., dairy products, fermented vegetables, fish and sausages—as well as silage inoculants [5,7,8]. *Lactobacillus rhamnosus* (*L. rhamnosus*) has been shown to produce lactic acid as the only carbohydrate metabolism product [9,10].

Lactic acid was first manufactured on a commercial scale in the United States in 1883 by lactic acid bacterial fermentation of sugar substrates [11]. Lactic acid bacteria of the genus *Lactobacillus* have been employed in manufacturing lactic acid. A natural organic acid with a long history of application in the food, leather, cosmetic, and pharmaceutical industries [12], lactic acid has also been described as a very effective exfoliating and moisturizing agent [13]. Its application at low concentration (5% v/v) decreases intercorneocyte cohesion and induces skin peeling [14]. Therefore, different concentrations of lactic acid produce different cosmetic results in the epidermis and dermis [15].

In the last few years, LAB were widely distributed in foods due to their beneficial effects on health, such increasing the innate immune response [16], helping to control intestinal infections [17], influencing cholesterol levels [18], antioxidant [19], and anticarcinogenic effects [20]. Some possible mechanisms of action maybe include the production of acid and other by-products of bacterial metabolism in the spent culture supernatant (SCS). For intestinal infections, SCS of the *L. rhamnosus* GG had been reported to exert antibacterial activity against *Salmonella typhimurium* [21]. *L. fermentum*-secreted compounds in SCS inhibit the growth, cytotoxicity and biofilm formation of several *S. aureus* and *P. aeruginosa* strains [22]. Three human-isolated *Lactobacillus* strains show antagonistic activity against ETEC infection *in vitro* [23]. SCS of *L. plantarum* 2142 can protect the response of enterocytes to oxidative stress from oxidative injury [24]. Although LAB strains have been shown to have wide applications, LAB culture filtrates are usually discarded and have not been studied in detail. There are no reports on the application of *L. rhamnosus* culture filtrate for use in cosmetic skin care products. The objective of this study was to investigate the effectiveness of *L. rhamnosus* spent culture supernatant (Lr-SCS) in cosmetics, with the aim of developing a possible new green cosmetic material.

## 2. Results and Discussion

### 2.1. DPPH Scavenging Activity Assay

**Figure 1:**
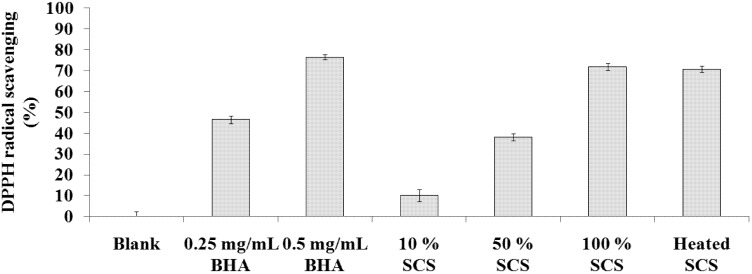
DPPH radical scavenging activities of standard samples and Lr-SCS samples.

DPPH is a stable free radical compound that has been widely used to measure the free radical-scavenging activity and hydrogen-donating ability of antioxidants. Figure 1 shows the DPPH radical scavenging activity of Lr-SCS samples increases in a dose-dependent manner. The radical scavenging activities for 10%, 50%, 100%, and heated SCS were 10.2 ± 2.9%, 38.1 ± 1.6%, 71.7 ± 1.7%, and 70.7 ± 1.5%, respectively. The DPPH radical-scavenging activities of BHA at 0.25 and 0.5 mg/mL were 46.4 ± 1.9% and 76.4 ± 1.3%, respectively. Moreover, the heated sample did not lose any DPPH scavenging activity. Heat treatment is usually needed to test the stability of a cosmetic product. The result of heat-stable Lr-SCS confirms this strain to be useful in the development of cosmetic materials.

### 2.2. ABTS^+^• Scavenging Capacity Assay

**Figure 2:**
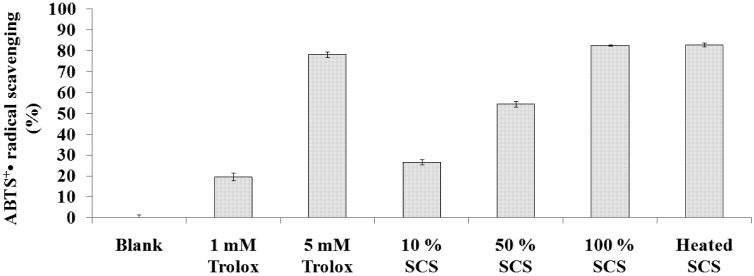
ABTS^+^• radical scavenging activities of standard samples and Lr-SCS samples.

We compared the efficacies of Lr-SCS samples and Trolox in scavenging the ABTS^+^• radical. Figure 2 illustrates that ABTS^+^• radicals were inhibited by 26.6 ± 1.3%, 54.5 ± 1.3%, 82.4 ± 0.3%, and 82.8 ± 0.9% by solutions of 10%, 50%, 100%, and heated Lr-SCS, respectively. The ABTS^+^• radical scavenging activities of Trolox at 1 and 5 mM were 19.7 ± 1.9% and 78.2 ± 1.4%, respectively. Heat treatment of Lr-SCS did not influence its ABTS^+^• scavenging activity, which was equivalent to that of 5 mM Trolox.

### 2.3. Determination of Reducing Power

**Figure 3:**
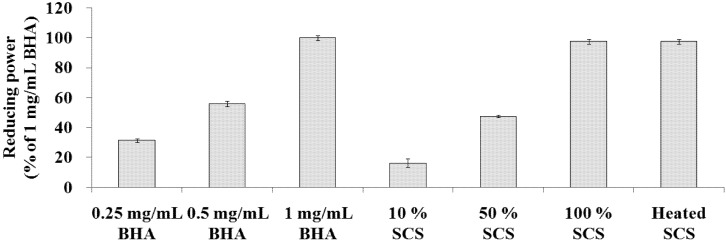
Reducing powers of standard samples and Lr-SCS samples.

The reducing power of Lr-SCS increased steadily with increasing concentration (Figure 3). In this study, the reducing power of samples was compared with that of 1 mg/mL BHA. The reducing powers for 10%, 50%, 100%, and heated SCS were 16.2 ± 2.7%, 47.4 ± 0.8%, 97.6 ± 1.6%, and 97.5 ± 1.7%, respectively. Meanwhile, the reducing power of 0.25 mg/mL BHA was 31.3 ± 1.3% and that of 0.5 mg/mL BHA was 55.8 ± 1.9%.

Qian *et al.* [25] showed that milk fermented with *Lactobacillus delbrueckii* ssp. *bulgaricus* LB340, after centrifugation and ultrafiltration, yielded peptides in the 3-5 kDa range that exhibited good antioxidant activity. Liu and Pan [26] indicated that heat-killed cells of *Lactobacillus acidophilus* BCRC 14079 exhibit high total antioxidant activity, which is probably related to a Maillard reaction, a reaction between sugar and amino acid in LAB culture after heating. Numerous research studies have focused on Maillard reaction products that may have some antioxidant properties, *i.e.*, clearance of hydroxyl radicals, clearance of hydrogen peroxide, and reduction of lipid peroxidation [27,28,29]. Liu *et al.* [30] demonstrated that *Lactobacillus* exopolysaccharides had antioxidant properties such as DPPH radical scavenging activity, chelation of ferrous ions, and reducing power *in vitro*. We speculate that the source of antioxidant activity in our study may similarly result from heat-stable peptides and/or Maillard-reaction products.

### 2.4. Tyrosinase Inhibitory Activity Assay

**Figure 4:**
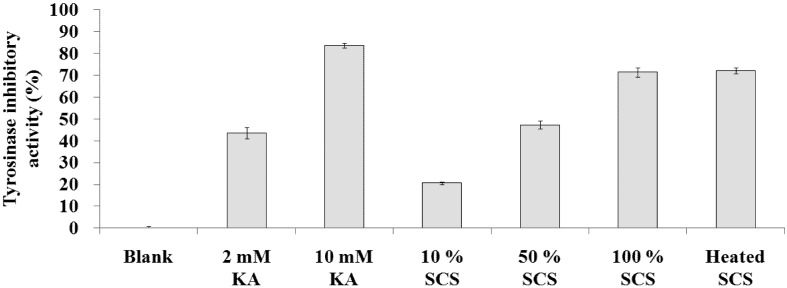
Tyrosinase inhibitory activities of standard samples and Lr-SCS samples.

The mushroom tyrosinase inhibitory activities for 10%, 50%, 100%, and heated Lr-SCS were 20.6 ± 0.7%, 47.2 ± 1.8%, 71.3 ± 2.2%, and 72.1 ± 1.2%, respectively (Figure 4). Meanwhile, the mushroom tyrosinase inhibitory activities for 2 and 10 mM kojic acid were 43.6 ± 2.5% and 83.6 ± 1.0%, respectively. Compared with untreated Lr-SCS, heated Lr-SCS did not appear to decrease tyrosinase inhibitory activity.

Usuki *et al.* [31] demonstrated that lactic acid could directly inhibit tyrosinase enzyme activity. However, this effect was not due to the acidity of lactic acid, because adjusting the pH did not affect inhibitory effect on tyrosinase activity. The results of this study were consistent with that report. In our study, the lactic acid in Lr-SCS, a major effective component, suppressed tyrosinase activity *in vitro* in a dose-dependent manner. Furthermore, heated Lr-SCS also directly inhibited tyrosinase activity.

### 2.5. Evaluation of Moisture Retention

**Figure 5:**
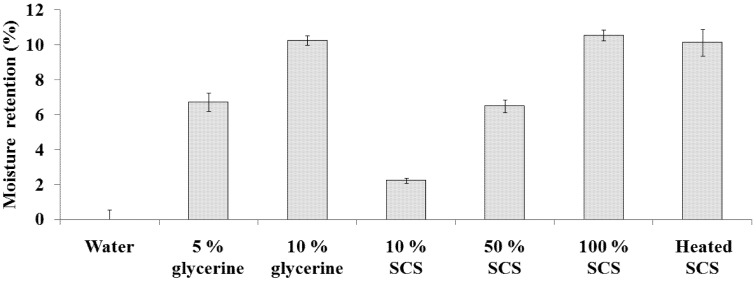
Moisture retentions of standard samples and Lr-SCS samples.

The *in vitro* moisture retention property of Lr-SCS was examined gravimetrically and compared with that of glycerin, frequently used as a hygroscopic and humectant agent. Figure 5 illustrates that moisture retention activities of 10%, 50%, 100%, and heated SCS were 2.2 ± 0.1%, 6.5 ± 0.4%, 10.5 ± 0.3%, and 10.1 ± 0.8%, respectively. Lr-SCS showed a dose-dependent trend in moisture retention, which was not influenced by heat treatment. In addition, moisture retention by Lr-SCS was equivalent to that of 10% glycerin. Table 1 summarizes the activities of Lr-SCS from Figure 1, Figure 2, Figure 3, Figure 4 and Figure 5.

**Table 1:** Activities of *Lactobacillus rhamnosus* spent culture supernatant.

	Sample	Blank	Control		SCS			Heated SCS
Activity			No. 1	No.2	10%	50%	100%	
DPPH scavenging (%)		0 ± 2.3	46.4 ± 1.8	76.4 ± 1.3	10.1 ± 2.9	38.0 ± 1.6	71.7 ± 1.7	70.6 ± 1.5
ABTS^+^• scavenging (%)		0 ± 1.4	19.6 ± 1.9	81.5 ± 1.6	26.6 ± 1.2	54.4 ± 1.3	82.4 ± 0.2	82.8 ± 0.8
Reducing power (%)			55.7 ± 1.9	100.0 ± 1.7	16.2 ± 2.7	47.4 ± 0.8	97.6 ± 1.6	97.4 ± 1.7
Tyrosinase inhibition (%)		0 ± 0.7	43.6 ± 2.5	83.5 ± 1.0	20.6 ± 0.7	47.1 ± 1.8	71.3 ± 2.2	72.0 ± 1.2
Moisture retention (%)		0 ± 0.5	6.7 ± 0.5	10.2 ± 0.2	2.2 ± 0.1	6.4 ± 0.3	10.5 ± 0.3	10.1 ± 0.7

### 2.6. Titratable Acidity and pH Measurement

**Figure 6:**
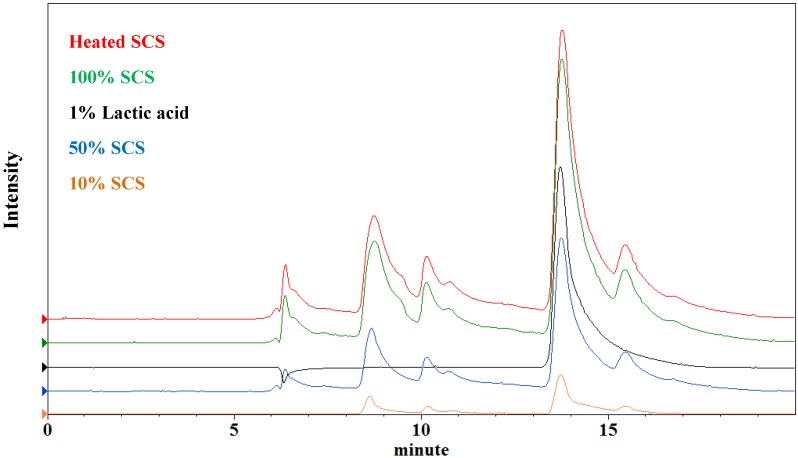
Intensity *vs.* retention time of lactic acid and Lr-SCS samples by HPLC.

The titratable acidity concentration and pH of 10%, 50%, 100%, and heated Lr-SCS were 0.16% (pH 4.3), 0.83% (pH 4.3), 1.65% (pH 4.3), and 1.64% (pH 4.3), respectively. As shown in Figure 6, the lactic acid concentrations by the HPLC method are 0.29%, 0.91%, 1.72%, and 1.68%, respectively. In this study, the pH of Lr-SCS was the same across various concentrations, as well in heated Lr-SCS. The lactic acid concentration of heated Lr-SCS was the same that of 100% Lr-SCS. It is presumed that the Lr-SCS has a good pH buffering capacity, probably deriving from the peptide fraction and resulting in the almost equal pH for different lactic acid concentrations. Lactic acid concentration was found to be correlated positively with moisture retention, in agreement with a previous report [13].

## 3. Experimental

### 3.1. Materials

The following chemicals were purchased from Sigma-Aldrich Co. (St. Louis, MO, USA): butylated hydroxyanisole (BHA); 1,1-diphenyl-2-picrylhydrazyl (DPPH); 2,2-azino-bis-(3-ethylbenzothiazoline-6-sulfonic acid) (ABTS); kojic acid; L-tyrosine; and mushroom tyrosinase. Iron (III) chloride was obtained from Riedel-de Haen (Seelze, Germany), and ethanol was purchased from Merck (Darmstadt, Germany).

### 3.2. Bacterial Culture

*Lactobacillus rhamnosus* LRH113 was obtained from Synbiotech Biotechnology (Kaohsiung, Taiwan). The bacterial strain was inoculated at 1% in 400 mL de Man Rogosa Sharp (MRS) broth (Difco, Detroit, MI, USA). After anaerobic incubation for 20 h at 37 °C (ThermoForma, Model 1025, Marietta, OH, USA), bacterial cells were removed by centrifugation (8,000 rpm, 10 min, 4 °C), and the supernatant was passed through a 0.45-µm cellulose acetate filter to obtain Lr-SCS. The samples tested in this study included Lr-SCS at various concentrations (10, 50, 100 v/v %) and heated Lr-SCS (100 °C for 30 min).

### 3.3. DPPH Scavenging Activity Assay

The DPPH scavenging activity of samples was measured according to Zhang’s method [32]. Lr-SCS (0.1 mL) and ethanol (0.4 mL) were added to DPPH (1 mL, 0.25 mM) solution. When DPPH reacts with an antioxidant that can donate hydrogen, it is converted to a reduced form and causes a decrease in absorbance at 517 nm (Sunrise ELISA Plate Reader, Tecan, Salzburg, Austria). In this study, BHA (0.25 and 0.5 mg/mL) was used as a standard. The percent DPPH scavenging activity was calculated using the formula: DPPH scavenging activity (%) = (1 − A_sample_/A_blank_) × 100, where A is the absorbance at 517 nm.

### 3.4. ABTS^+^• Scavenging Capacity Assay

The ABTS^+^• scavenging capacity assay was carried out using the procedure described in Erkan’s method [33]. In brief, ABTS^+^• was produced by reacting 7 mM ABTS with 2.45 mM potassium persulfate in the dark for 16 hours at 26 °C. A 10-μL Lr-SCS sample was added to 2 mL ABTS^+^• radical solution, allowed to react for 10 min, and the absorbance was measured at 734 nm. The ABTS^+^• scavenging capacity of the sample was compared with that of Trolox (6-hydroxy-2,5,7,8-tetramethylchroman-2-carboxylic acid), which is a vitamin E derivative with potent antioxidant properties and commonly used as a standard or positive control in antioxidant assays. ABTS^+^• scavenging activity was calculated using the formula: ABTS^+^• scavenging activity (%) = (1 − A_sample_/A_blank_) × 100, where A is the absorbance at 734 nm.

### 3.5. Determination of Reducing Power

The reducing power of each Lr-SCS sample was determined using a mixture of phosphate buffer (1 mL, 0.2 M, pH 6.6), potassium ferricyanide (1 mL, 1% by weight), and sample (1 mL) [34]. The mixture was then incubated for 20 minutes at 50 °C. A volume of trichloroacetic acid (1 mL, 1% by weight) was added to the mixture, which was then centrifuged at for 10 min at 3,000 rpm. The upper layer of solution (0.4 mL) was mixed with distilled water (0.5 mL) and FeCl_3_ (1 mL, 0.1% by weight) for 10 min, and the absorbance was measured at 700 nm. A higher absorbance of the reaction mixture was indicative of greater reducing power. Reducing power was calculated using the formula: Reducing power (%) = (A/B) × 100, where A is the sample absorbance and B is the absorbance of 1 mg/mL BHA.

### 3.6. Tyrosinase Inhibitory Activity Assay

Tyrosinase inhibitory activity was measured using the method described by Jo [35]. An aqueous solution of mushroom tyrosinase (2 μL, 2 units) was added to a 96-well microplate, following by a mixture (120 μL) containing 5 mM L-dopa dissolved in 67 mM phosphate buffer (pH 6.8) and a Lr-SCS sample (20 μL). The assay mixture was incubated for 30 minutes at 37 °C. Following incubation, the amount of dopachrome produced in the reaction mixture was determined by spectrophotometric analysis at 490 nm. In this study, kojic acid (10 and 2 mM) was used as a standard. Percent inhibition of tyrosinase activity was calculated using the formula: Inhibition (%) = (1 − OD_sample_/OD_blank_) × 100, where OD is optical density at 490 nm.

### 3.7. Evaluation of Moisture Retention

The weight loss of a sample was used for evaluation of moisture retention. A Lr-SCS sample (900 µL) was placed in a glass tube and transferred for 96 hours to an oven with constant temperature and humidity (50 °C, 60% relative humidity). The weights before (W_0_) and after (W_t_) the incubation were recorded by an electronic balance (XS 225A, Precisa Gravimetrics AG, Dietikon, Switzerland). In this study, glycerin was used as a moisture retention standard because it is a well-known cosmetic ingredient, and it exists in the stratum corneum as a natural endogenous humectant [36]. The moisture retention rate (Rr) was evaluated by the weight loss of the sample: Rr (%) = [1 − (W_0,sample_ − W_t,sample_)/(W_0,control_ − W_t,control_)] × 100.

### 3.8. Titratable Acidity and pH Measurement

Titratable acidity was assessed by the titration method of Nwafor and Ikenebomeh [37]. A Lr-SCS sample (approximately 10 g) was mixed with 0.5% (w/v ethanol) phenolphthalein solution (0.5 mL) and titrated by 0.1 N NaOH until a faint pink color persisted. The pH was measured using a pH-meter (SP-2300, Suntex Instruments Company Ltd, Taichung, Taiwan). Percent lactic acid was determined using the formula: LA % = 100 × (0.1 N NaOH required × 0.1 N NaOH factor × 0.009) ÷ (sample weight). High performance liquid chromatography (HPLC) analysis on lactic acid content was performed on a Hitachi L-7100 system (Merck-Hitachi, Darmstadt, Germany) equipped with a RI detector and an organic acid column (Rezex ROA H^+^ form, 5 μm, 300 × 7.8 mm I.D.; Phenomenex, Torrance, CA, USA). The flow rate was 0.6 mL/min with a solvent of 0.005 N H_2_SO_4_ at 75 °C.

## 4. Conclusions

This is the first report that has evaluated *Lactobacillus rhamnosus* spent culture supernatant for cosmetic use. We examined six assays: DPPH scavenging activity, ABTS^+^• scavenging capacity, reducing power, tyrosinase inhibitory activity, moisture retention, and titratable acidity. Lr-SCS showed dose-dependent increases in lactic acid concentration, antioxidant activity, inhibitory tyrosinase synthesis, and moisture retention. Heat treatment did not destroy the active components of Lr-SCS for these assays. In the future, we will further analyze the active components in Lr-SCS and elucidate the antioxidant and whitening mechanisms for skin care cosmetics.
